# Comparison of clinical and radiographic outcomes for platform-switched bone-level and platform-matched tissue-level implants

**DOI:** 10.6026/973206300214756

**Published:** 2025-12-15

**Authors:** Himaja Kurlagunda, Rajesh Nichenametla, Ramesh Amirisetty, Parthasaradhi T, Nandini R, Neha Singh

**Affiliations:** 1 Periodontist, Practitioner in Hyderabad, India; 2 Department of Periodontology, G. Pulla Reddy Dental College And Hospital, Kurnool, Andhra Pradesh, India; 3 Department of Periodontology, Tagore Dental College And Hospital, Rathinamangalam, Melakkattiyur, Tamilnadu, India

**Keywords:** Dental implants, tissue-level implants, bone-level implants, platform switching, crestal bone loss, CBCT

## Abstract

Crestal bone preservation is essential for dental implant longevity, yet evidence comparing tissue-level (TL) and bone-level (BL) designs remains inconsistent. Therefore, it is of interest to evaluate 30 implant sites assigned to TL (n=15) or platform-switched BL (n=15) implants to compare clinical outcomes, crestal bone levels, and implant stability. Clinical parameters including bleeding on probing, plaque index, probing depth, gingival margin position, and clinical attachment level were recorded at 2 and 8 months, while CBCT assessed crestal bone changes and resonance frequency analysis measured implant stability. Both groups showed comparable peri-implant soft-tissue parameters and similar crestal bone loss at 8 months, with no significant differences in implant stability. Both TL and BL implant designs demonstrated equivalent clinical and radiographic performance, confirming their suitability for implant-supported restorations.

## Background:

Dental implants have evolved significantly since their introduction, becoming a standard treatment modality for replacing missing teeth [1]. The success of implant therapy depends on various factors, with maintenance of peri-implant bone being one of the most critical determinants of long-term outcomes [2]. Crestal bone loss around dental implants has been traditionally accepted at approximately 1.5 mm during the first year and 0.2 mm annually thereafter [3]. However, recent studies emphasize that maintaining the initially achieved peri-implant bone levels as coronally as possible is crucial for long-term success and optimal aesthetic results [4]. The two primary implant designs currently available are tissue-level (TL) and bone-level (BL) implants. TL implants, also known as transmucosal implants, feature a smooth collar that extends above the bone crest, allowing the soft tissue to attach directly to the implant body. This design was popularized by the ITI Dental Implant System in the 1970s and has been extensively documented in the literature [5]. In contrast, BL implants are designed to be placed at the level of the alveolar bone crest, with the implant-abutment connection positioned at or near the bone level [6]. The concept of platform switching was accidentally discovered in the late 1980s and has since gained significant attention in implant dentistry [7]. This approach involves using an abutment with a smaller diameter than the implant platform, creating a horizontal mismatch that may help preserve crestal bone by positioning the inflammatory cell infiltrate away from the bone [8]. Several hypotheses have been proposed to explain the benefits of platform switching, including horizontal repositioning of the biologic width, increased distance between inflammatory cells and bone crest, and improved stress distribution at the implant-abutment interface [9]. Crestal bone resorption around BL implants has been attributed to various factors, including surgical trauma, establishment of biologic width, micromovement at the implant-abutment interface, and bacterial colonization in the microgap area [10]. TL implants were designed to address these issues by positioning the microgap away from the bone crest, potentially reducing bacterial contamination and subsequent inflammation [11]. However, long-term radiographic studies of TL implants have also demonstrated peri-implant bone loss, ranging from 0.6 to 1.0 mm during the first year of function [12]. Two-dimensional radiographic techniques, such as intraoral radiographs and panoramic imaging, have limitations in evaluating peri-implant bone levels due to anatomical superimposition and distortion [13]. Cone beam computed tomography (CBCT) has emerged as a valuable tool for three-dimensional assessment of peri-implant bone, providing superior image quality for dento-maxillofacial applications and hard-tissue visualization compared to conventional radiography [14]. Despite numerous studies comparing TL and BL implants, conflicting observations regarding marginal bone loss have been reported [15, 16]. Some studies suggest that TL implants may better preserve crestal bone due to the microgap being positioned away from the bone [17], while others report no significant differences between the two designs [18]. Therefore, it is of interest to prospectively compare the clinical and radiographical outcomes of platform-switched BL implants and platform-matched TL implants using CBCT for accurate three-dimensional assessment.

## Materials and Methods:

## Study design:

This prospective clinical study was conducted at the Department of Periodontics and Implantology, G. Pulla Reddy Dental College & Hospital, Kurnool.

## Sample size:

A total of 30 edentulous sites in patients aged 18-50 years were included in the study. Sample size was determined based on previous similar studies, with 15 sites randomly assigned to each group: Group I received TL implants, while Group II received BL implants.

## Inclusion criteria:

[1] Patients aged 18-50 years

[2] Single or multiple missing teeth with healed sites (≥6 months)

[3] Implant site free from infection and extraction remnants

[4] Adequate bone height (≥12 mm) and width (≥6 mm)

[5] Good oral hygiene and compliance with follow-up visits

## Exclusion criteria:

[1] Systemic diseases that could affect wound healing

[2] Smoking or tobacco chewing habits

[3] Need for bone augmentation procedures

[4] Pregnant or lactating women

[5] Patients undergoing radiation or immunosuppressive therapy

[6] Bone metabolic diseases or disorders

[7] Trauma from occlusion and malocclusions

[8] HIV or hepatitis positivity

## Materials:

OSSTEM implants (FDA-approved) were used in this study. For Group I, OSSTEM SS system implants (tissue-level) with internal octa connection were selected, featuring a taper body, wide crown margin, corkscrew thread, and helix cutting edges. For Group II, OSSTEM TS system implants (bone-level) with internal hex platform switching were used, featuring a taper body, helix cutting edges, and corkscrew thread. Implant dimensions ranged from 3.0-7.0 mm in diameter and 6.0-15.0 mm in length, as determined by preoperative CBCT measurements. Straight titanium abutments were used for Group I, while COM octa titanium abutments were used for Group II. All final restorations were all-ceramic crowns.

## Surgical procedure:

All patients underwent comprehensive preoperative assessment including detailed case history, clinical examination, and routine blood investigations. Oral prophylaxis was performed, and oral hygiene instructions were provided. Alginate impressions were taken for both arches, and diagnostic casts were articulated to evaluate occlusion. Preoperative CBCT (Carestream®) was used to assess the position of anatomical structures and determine implant position, length, and diameter, ensuring at least 2 mm from the mandibular canal and preserving 1.5 mm of peri-implant bone. Surgical stents were fabricated for each patient. All surgical procedures were performed under local anesthesia (2% lignocaine with 1:80,000 adrenaline). A mid-crestal incision was made using a #15 surgical blade, preserving at least 2 mm of keratinized tissue on the buccal side. Sulcular incisions were made at adjacent teeth, followed by elevation of full-thickness mucoperiosteal flaps buccally and lingually. Crestotomy was performed at the intended osteotomy site to minimize irregularities. The implant site was prepared according to the manufacturer's instructions using a physio-dispenser set at 850 RPM with torque of approximately 35 Ncm. The surgical stent was positioned, and initial drilling was performed with a pilot drill. Sequential drilling was carried out, followed by thorough irrigation of the implant bed with saline. OSSTEM implants with appropriate length and diameter were placed into the osteotomy site. In Group I, implants were positioned exactly at the junction between the rough and smooth surface at the crestal bone level. In Group II, implants were placed at the crestal bone level. Insertion torque was maintained between 30-35 Ncm using a torque wrench. Primary stability was assessed using both the torque wrench and PENGUIN RFA (Resonance Frequency Analysis). Cover screws were placed, and primary flap closure was achieved using direct loop suturing with 3-0 Ethicon black braided silk suture material. No graft materials were used in any surgical sites, and no temporary prostheses were placed to avoid inadvertent trauma.

## Postoperative care and evaluation:

Patients were advised to consume only soft and semi-solid foods for the first three days postoperatively. All patients received broad-spectrum antibiotics and appropriate analgesics for five days. Chlorhexidine mouthwash (0.2%) was prescribed twice daily for four weeks. Sutures were removed after seven days, and patients were recalled monthly for review.

The operated area was evaluated for healing, infection, and signs of ulceration or necrosis. Postoperative maintenance was provided monthly in both groups.

## Prosthodontic phase:

After two months of implant placement, patients were recalled for the prosthodontic phase. Implant-level impressions were taken using silicone putty material followed by soft body material. Casts were prepared and articulated, and all-ceramic crowns were fabricated in the laboratory. Healing abutments were removed, and crowns were fixed using composite cement. Patients underwent CBCT immediately after crown placement.

## Follow-up and evaluation parameters:

Patients were followed up at two and eight months postoperatively. The following parameters were evaluated:

## Clinical parameters:

[1] Bleeding on probing (modified Sulcus Bleeding Index by Mombelli)

[2] Plaque index (Silness & Löe)

[3] Peri-implant probing depth

[4] Relative gingival margin position

[5] Relative clinical attachment level

## Radiological parameters:

Crestal bone levels were measured using CBCT at baseline, two months, and eight months. Using Carestream® software, oblique slicing was performed to locate the edentulous gap in the axial plane, and sections were obtained in sagittal and coronal planes. The long axis of the implant was identified, and a tangent was drawn perpendicular to this axis. The distance from the implant shoulder to the most coronal crestal bone in contact with the implant was assessed on buccal, lingual, mesial, and distal aspects.

## Implant stability:

Implant stability was evaluated using PENGUIN RFA at baseline and two months, with measurements recorded as Implant Stability Quotient (ISQ) values.

## Statistical analysis:

Statistical analysis was performed using appropriate tests. Intragroup comparison of clinical parameters at two and eight months was done using the Wilcoxon signed-rank test. Intergroup comparison of periodontal parameters based on mean difference from two to eight months was performed using the Mann-Whitney U test. Intragroup comparison of crestal bone levels at different intervals was evaluated using the Friedman test. For implant stability, intragroup comparison was made using the paired t-test, while intergroup comparison was done using the unpaired t-test. A p-value <0.05 was considered statistically significant.

## Results:

In Group I (TL implants), the mean bleeding on probing score increased from 0.47±0.52 at two months to 0.80±0.77 at eight months. In Group II (BL implants), the mean score increased from 0.53±0.52 to 0.93±0.70. The difference between groups was not statistically significant (p=0.792). Both groups showed a statistically significant increase in plaque index from two to eight months. In Group I, the mean value increased from 0.41±0.21 to 0.88±0.48 (p=0.002), while in Group II, it increased from 0.53±0.23 to 0.91±0.38 (p=0.002). However, the difference between groups was not significant (p=0.868). In Group I, the mean probing depth decreased from 1.87±0.74 mm at two months to 1.67±0.62 mm at eight months. In Group II, it increased from 1.93±0.88 mm to 2.2±0.68 mm. The difference between groups was not statistically significant (p=0.162). In Group I, the mean relative gingival margin position remained unchanged at 6.73±0.80 mm at both time points. In Group II, it increased slightly from 6.8±0.86 mm to 6.93±0.96 mm. The difference between groups was not statistically significant (p=0.164). In Group I, the mean relative clinical attachment level decreased from 8.60±1.06 mm at two months to 8.47±1.06 mm at eight months. In Group II, it increased from 8.53±0.92 mm to 9.13±0.92 mm. The difference between groups was statistically significant (p=0.024). In Group I, the mean crestal bone levels showed significant changes at all aspects (mesial, distal, buccal, lingual) from baseline to eight months (p<0.001). Similarly, in Group II, significant changes were observed at all aspects (p<0.001) (Table 1 - see PDF, 2 - see PDF).

The mean crestal bone loss from baseline to eight months showed no statistically significant differences between groups at any aspect:

[1] Mesial aspect: 0.64±0.238 mm (Group I) vs. 0.647±0.164 mm (Group II); p=0.643

[2] Distal aspect: 0.547±0.188 mm (Group I) vs. 0.607±0.22 mm (Group II); p=0.401

[3] Buccal aspect: 0.713±0.146 mm (Group I) vs. 0.747±0.155 mm (Group II); p=0.539

[4] Lingual aspect: 0.580±0.214 mm (Group I) vs. 0.553±0.210 mm (Group II); p=0.673

In Group I, the mean ISQ value increased from 77.5±6.76 at baseline to 80.1±4.72 at two months. In Group II, it increased from 77.3±7.13 to 78.9±4.33. Both groups showed improved stability at two months compared to baseline. No statistically significant differences were observed between groups at baseline (p=0.958) or at two months (p=0.474) (Table 3 - see PDF).

## Discussion:

This prospective study compared clinical and radiographical outcomes of platform-matched TL implants and platform-switched BL implants over an 8-month period. The results demonstrated no statistically significant differences between the two implant types in most clinical parameters, crestal bone changes, or implant stability, suggesting that both designs may be equally effective in the short to medium term. The maintenance of peri-implant bone health is crucial for the long-term success of dental implants [19]. Crestal bone loss has been associated with various factors, including surgical trauma, occlusal overload, peri-implantitis, microgap at the implant-abutment interface, and establishment of biologic width [20]. In the present study, both TL and BL implants showed minimal crestal bone loss (<1 mm) at all aspects (mesial, distal, buccal, lingual) over the 8-month observation period, which is within the acceptable range reported in the literature [21]. The concept of platform switching was introduced to potentially reduce crestal bone loss by positioning the inflammatory cell infiltrate away from the bone crest [22]. However, in our study, BL implants with platform switching did not show superior crestal bone preservation compared to TL implants with platform matching. This finding is consistent with previous studies that reported no significant differences in marginal bone loss between TL and BL implants [23, 24]. The similar bone loss patterns observed in both groups may be attributed to several factors, including the use of implants with SLA surfaces, controlled surgical protocols, and adequate primary stability. Clinical parameters such as bleeding on probing, plaque index, and probing depth are important indicators of peri-implant tissue health [25]. In our study, both groups showed an increase in plaque index from 2 to 8 months, which was statistically significant. This increase in plaque accumulation may be attributed to patient compliance with oral hygiene instructions despite regular reinforcement. The significant increase in plaque index could explain the slight increase in bleeding on probing scores observed in both groups, although this difference was not statistically significant. The relative clinical attachment level showed a statistically significant difference between groups, with TL implants demonstrating a slight decrease and BL implants showing an increase over the observation period. This finding may suggest better soft tissue stability around TL implants, possibly due to the transmucosal design allowing for better soft tissue adaptation [26]. However, the clinical significance of this difference remains uncertain, as both groups maintained healthy peri-implant tissues throughout the study. Implant stability is a critical factor for successful osseointegration [27]. In our study, both groups showed an increase in ISQ values from baseline to 2 months, indicating improved secondary stability. This finding is consistent with previous studies that reported an initial decrease in stability followed by an increase as bone healing progresses [28]. The similar stability values between groups suggest that both implant designs can achieve adequate primary and secondary stability when properly placed. The use of CBCT for evaluating crestal bone levels provided a comprehensive three-dimensional assessment of bone changes around the implants [29]. This imaging modality allowed for precise measurements at all aspects (mesial, distal, buccal, lingual), overcoming the limitations of conventional two-dimensional radiography [30]. The greater bone loss observed on the buccal aspect in both groups may be attributed to the denser and less vascular nature of the buccal bone plate compared to interdental or lingual bone [31]. Several factors may have contributed to the favorable outcomes observed in both groups. The use of a conservative surgical approach with proper flap reflection, controlled drilling speed and torque, and avoidance of countersink drills may have minimized surgical trauma to the crestal bone [32]. Additionally, the use of SLA surfaced implants and appropriate abutment connections may have promoted favorable tissue response [33]. The 8-month follow-up period in our study is relatively short, and longer-term studies are needed to evaluate the stability of these results over time. Previous studies have suggested that differences between TL and BL implants may become more apparent with longer observation periods [34]. Furthermore, the sample size of 30 sites, while adequate for detecting significant differences in most parameters, may have limited the statistical power to detect smaller differences between groups. Despite these limitations, our findings suggest that both platform-matched TL implants and platform-switched BL implants can achieve favorable clinical and radiographical outcomes in the short to medium term. TL implants may offer certain advantages in specific clinical situations, such as cases with limited keratinized tissue or when a simplified surgical approach is desired [35]. However, the choice between TL and BL implants should be based on individual patient factors, clinician experience, and specific clinical requirements.

## Conclusion:

Both platform-matched tissue-level implants and platform-switched bone-level implants showed similar clinical and radiographical outcomes over an 8-month observation period. No statistically significant differences were observed between the two implant types in bleeding on probing, plaque index, probing depth, relative gingival margin position, crestal bone changes at all aspects, or implant stability. A significant difference was only noted in relative clinical attachment level, with tissue-level implants showing slightly better outcomes. Both implant designs demonstrated minimal crestal bone loss (<1 mm) and favorable soft tissue parameters, suggesting that either type can be a viable option for implant-supported restorations. Tissue-level implants may offer certain advantages in specific clinical situations, such as cases requiring a simplified surgical approach or when preservation of soft tissue architecture is a priority. However, longer-term studies with larger sample sizes are needed to confirm these findings and evaluate the stability of the results over extended periods.
